# Microbiome Markers in Gastrointestinal Disorders: Inflammatory Bowel Disease, Colorectal Cancer, and Celiac Disease

**DOI:** 10.3390/ijms26104818

**Published:** 2025-05-17

**Authors:** M. Isabel San-Martin, Alejandro Chamizo-Ampudia, África Sanchiz, Miguel Ángel Ferrero, Honorina Martínez-Blanco, Leandro Benito Rodríguez-Aparicio, Nicolás Navasa

**Affiliations:** 1 Area Biochemistry, Department of Molecular Biology, University of León, 24071 León, Spain; msanb@unileon.es (M.I.S.-M.); alejandro.chamizo@unileon.es (A.C.-A.); ma.ferrero@unileon.es (M.Á.F.); hmarb@unileon.es (H.M.-B.); nnavm@unileon.es (N.N.); 2 Instituto de Biología Molecular, Genómica y Proteómica (INBIOMIC), Universidad de León, Campus de Vegazana, 24071 León, Spain

**Keywords:** metagenomic, microbiota, bowel disease, inflammation, biomarkers

## Abstract

Intestinal microbiota and the host’s immune system form a symbiotic alliance that sustains normal development and function in the human gut. Changes such as dietary habits among societies in developed countries have led to the development of unbalanced microbial populations in the gut, likely contributing to the dramatic increase in inflammatory diseases in the last few decades. Recent advances in DNA sequencing technologies have tremendously helped to characterize the microbiome associated with disease, both in identifying global alterations and discovering specific biomarkers that potentially contribute to disease pathogenesis, as evidenced by animal studies. Beyond bacterial alterations, non-bacterial components such as fungi, viruses, and microbial metabolites have been implicated in these diseases, influencing immune responses and gut homeostasis. Multi-omics approaches integrating metagenomics, metabolomics, and transcriptomics offer a more comprehensive understanding of the microbiome’s role in disease pathogenesis, paving the way for innovative diagnostic and therapeutic strategies. Unraveling the metagenomic profiles associated with disease may facilitate earlier diagnosis and intervention, as well as the development of more personalized and effective therapeutic strategies. This review synthesizes recent and relevant microbiome research studies aimed at characterizing the microbial signatures associated with inflammatory bowel disease, colorectal cancer, and celiac disease.

## 1. Introduction

The intestinal microbiota is a group of microorganisms, including bacteria, viruses, and fungi, which inhabit the human gut and provide essential functions for human health [1]. For example, the intestinal microbiota participates in promoting immune cell homeostasis, maintaining epithelial function, providing defenses against harmful microorganisms, or contributing to host metabolism [2]. Changes in the lifestyle of human populations, such as their dietary habits or the overuse of antibiotics, may alter the composition and proportions of microbial populations (called “dysbiosis”) in the gut and disrupt the balance of communication between the microorganisms and the host [3]. These events are identified to partially explain the rapid increase in autoimmune and inflammatory diseases seen in developed countries [3]. Thus, microbial communities or specific taxa may be both the cause and/or consequence of disease development. Under this scenario, it may be that global microbial profiles or specific taxa are representative of disease or may even reflect a specific disease stage, which collectively situates the microbiota as both a potential biomarker and a factor contributing to human inflammatory diseases [4]. A deeper knowledge of the microbial signatures associated with disease may provide a useful predictive tool for earlier diagnosis and may help to design more efficient preventive or therapeutic strategies targeting microbiota [5].

Next-generation sequencing is a high-throughput technology that offers massive sequencing capability and allows researchers to characterize more precisely the taxonomic composition of microbial communities in health and disease. Many of these microbiome research studies are based on the sequencing of specific variable regions, such as the 16S ribosomal RNA (rRNA) gene for bacteria, which is cost-effective and has proven useful for broad taxonomic surveys, and 18S rRNA or its internal transcribed spacer for fungi [6]. However, the characterization of the microbiomes associated with some gastrointestinal diseases has recently improved tremendously, due to the combination of two main approaches. The arrival of shotgun technology for the direct sequencing of DNA has enabled more precise taxonomic identification of microbial markers, as well as improving the detection of low-abundance microorganisms and analyzing microbial gene function [7]. In addition, recent studies have included a higher number of patients who are often from different populations in terms of their geographical origin or sociocultural habits, which provides more reliable and universal biomarkers [8,9]. In this review, we try to synthesize recent advances derived from microbiome research studies that have contributed to identifying the alterations in bacteria, viruses, and fungi in microbiomes that are associated with inflammatory bowel disease (IBD), colorectal cancer (CRC), and celiac disease (CeD).

## 2. The Gut Microbiome in Inflammatory Bowel Disease

Since the 20th century, the incidence and prevalence of IBD have increased, particularly in industrialized countries, with more recent rises observed in newly industrialized regions. This global trend suggests that both genetic predisposition and environmental factors—such as diet, smoking, and medication—contribute to disease susceptibility by altering the gastrointestinal epithelium and modulating the intestinal microbiota.

The pathogenesis of IBD has been strongly associated with disruptions in the intestinal microbiota. Elevated levels of pathogenic bacteria, including *Escherichia coli* [10,11] and *Klebsiella* [12,13], alongside reduced levels of beneficial microbes such as *Clostridium* species [14,15] and *Bacteroides fragilis* [16,17] have been reported. Viruses and fungi also play significant roles; certain viruses have demonstrated protective effects against inflammation [18,19], while others, such as norovirus, have been shown to exacerbate it [20,21]. Fungal imbalances—such as an increased abundance of *Malassezia restricta* [22] with the absence of *Saccharomyces cerevisiae*—further contribute to disease progression [23,24].

**Figure 1:**
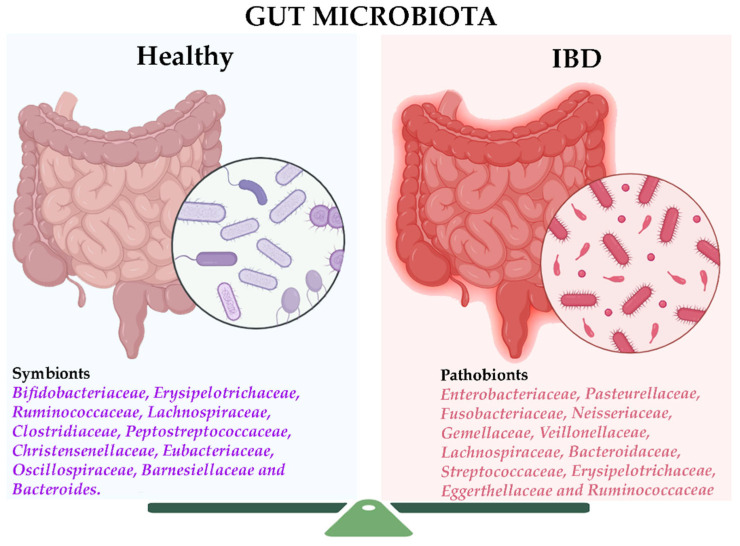
Changes in the intestinal microbiota of patients with inflammatory bowel disease (IBD) are characterized by a decrease in diversity, a reduction in probiotics, and an increase in pathogenic bacteria, which contribute to the onset of intestinal inflammation (modified figure derived from [28] and created in https://BioRender.com).

These findings support the hypothesis that IBD results from a complex interplay among genetic susceptibility, environmental exposure, and microbial dysbiosis, with immune dysregulation perpetuating chronic intestinal inflammation [25,26,27]. A deeper understanding of these mechanisms is essential for identifying biomarkers and developing targeted therapeutic strategies (Figure 1).

### 2.1. Bacterial Markers in IBD

In early studies based on 16S rRNA amplicon sequencing, alterations in bacterial composition and abundance in the intestines of patients with IBD were identified [29,30,31]. Subsequently, investigations using shotgun sequencing technologies and larger, more diverse cohorts have been conducted. These studies have enabled a more in-depth characterization of the bacterial microbiome associated with IBD in both pediatric [32,33,34] and adult populations [8,35,36,37,38,39,40]. As a result of these advances, previously described bacterial markers have since been confirmed, and new indicators have been identified (Table 1).

IBD has been associated with a loss of bacterial diversity, along with an increase in potentially pathogenic bacteria, such as the families of *Bacteroidaceae* and *Enterobacteriaceae*, as well as the species *Fusobacterium*. Conversely, a decrease in beneficial commensal bacteria, primarily from the families *Lachnospiraceae*, *Clostridiaceae*, and *Ruminococcaceae*, as well as certain species of *Bacteroides* and *Bifidobacterium* has been observed [43,44,45,46,47].

Various studies have suggested microbial differences between Crohn’s disease (CD) and ulcerative colitis (UC). Analyses based on fecal samples [35,36,48], biopsies [39,49], and endoscopic brushes [5,38] have shown greater dysbiosis in CD patients compared to controls, whereas microbial signatures in UC are more similar to those in healthy individuals. In contrast, some studies have reported comparable dysbiotic profiles between both IBD subtypes and controls [37].

Dysbiosis in CD has been characterized by a significant enrichment of facultative anaerobes, such as *Escherichia coli* [8,35,36,50]. Additionally, other over-represented taxa have been identified, including *Fusobacterium* [31,35,51], *Ruminococcus gnavus*, *Clostridium clostridioforme* [36,52,53], and *Ruminococcus torques* [8,54]. In parallel, a reduction in beneficial taxa such as *Faecalibacterium* has been described compared to those in UC, as well as in the families *Peptostreptococcaceae* and *Christensenellaceae*, and in the genera *Anaerostipes*, *Methanobrevibacter*, and *Collinsella* [35,55,56]. Similarly, species such as *Bifidobacterium breve*, *Clostridium symbiosum* [36], and *Roseburia hominis* have shown reduced abundance in CD [8].

In studies using endoscopic brushes, a greater abundance of *Escherichia*, *Clostridium*, *Cetobacterium*, *Peptostreptococcus*, and *R. gnavus* has been reported in the mucosa of CD patients, whereas *Faecalibacterium*, *Blautia*, *Bifidobacterium*, and *Roseburia* have been reported as enriched in UC [38,57]. In pediatric populations, the microbiomes of children with newly diagnosed CD [41,58] and UC [33,59] have shown a higher abundance of pathogenic taxa, especially bacteria from the upper respiratory tract, along with a reduction in the beneficial microbes primarily belonging to the *Clostridiales* group (*Lachnospiraceae* and *Ruminococcaceae*) [60,61,62].

Oxidative stress associated with intestinal inflammation in IBD has been linked with a shift from a predominantly obligate anaerobic microbiome to an aerotolerant one. This shift has also been reflected in the metabolic capacity of the microbiome. In line with this finding, functional analyses derived from metagenomic data have shown significant alterations in metabolic pathways in the inflamed intestine [36,37,41,42,63], including:

(i) An increase in redox tolerance [25,44,46,64,65];
(ii) A decrease in carbohydrate fermentation pathways (primarily those associated with *Clostridia* clades IV and XIVa) [45,64,66,67,68];
(iii) Maintenance of intestinal barrier function [45,64,69];
(iv) A reduction in pathways related to vitamin and amino acid synthesis [70,71];
(v) A relevant role in inflammation-associated immunosuppression [25,45,72]; and
(vi) An increase in virulence factors and antibiotic resistance, possibly linked to the rise of *Enterobacteriaceae* and *Bacteroides*.

Enterotoxigenic *Escherichia coli* (ETEC) plays a key role in gastrointestinal disorders, particularly through its enterotoxins, heat-labile toxin (LT) and heat-stable toxin (ST), which disrupt the fluid and electrolyte balance by targeting intestinal enterocytes [73,74]. Other exotoxins like EAST-1 and hemolysins worsen disease severity by compromising the intestinal barrier [75]. In contrast, short-chain fatty acid (SCFA)-producing bacteria, such as those producing acetate, propionate, and butyrate, support intestinal barrier integrity and modulate inflammation, which is critical in IBD [76,77]. These bacteria influence immune responses and enhance epithelial cell functions, making them crucial for maintaining gut health [78].

Bacterial virulence factors can also disrupt the intestinal epithelial barrier, exacerbating IBD symptoms. Toxins like *Clostridium perfringens* enterotoxin (CPE) and *Vibrio cholerae zonula occludens* toxin (ZOT) impair tight-junction proteins, increasing their permeability [79]. Bile acids (BAs), which modulate immune responses and microbiota composition, are imbalanced in IBD, contributing to inflammation by altering primary and secondary BA ratios [80]. Additionally, the microbial metabolism of tryptophan into bioactive indoles like indole-3-acetic acid (IAA) and indole-3-propionic acid (IPA) plays a role in immune modulation and intestinal homeostasis, with certain bacteria influencing these pathways [81,82].

### 2.2. Non-Bacterial Markers in IBD

A previous study on the virome, based on the DNA-based enumeration of virus-like particles (VLPs), reported a significant increase in the number of bacteriophages in the mucosa of patients with CD compared to controls [83]. Bacteriophages, specific parasites of bacteria, are critically involved in the stability of bacterial communities and the composition of the gut microbiome, with the most representative groups being *Caudovirales*, *Microviridae*, *crAssphages*, and *Gubaphages* [84,85,86,87]. Recent studies employing DNA sequencing from VLP preparations have further characterized the virome associated with IBD in both murine models [88] and human subjects [21].

In patients with IBD, the intestinal virome has been characterized by an increased richness of *Caudovirales* bacteriophages, and specific differences between CD and UC have been observed in terms of bacteriophage diversity and composition [84,85,86,87]. Interestingly, these variations do not appear to correlate directly with changes in bacterial composition. In UC, an increased abundance of *Caudovirales* in the mucosa has been identified compared to controls, although this is accompanied by a decrease in the diversity, evenness, and richness of both *Caudovirales* and viruses in general [89,90]. However, phages may confer acid pH resistance to pathogenic bacteria, as observed in *E. coli* when infected with bacteriophage Φ24 [85].

In contrast, no significant differences in the virome were found by Lewis et al. when fecal samples from patients with CD and controls were analyzed [42,91]. A possible explanation lies in the methodology employed, as VLPs were sequenced from whole stool samples without prior purification, which may have limited the precise identification of specific viral communities.

Regarding the mycobiome, studies on fecal samples [92,93,94] and biopsies [95,96] from patients with CD have evidenced fungal dysbiosis, characterized by an increased abundance of fungi, particularly species of the *Candida* genus (such as *C. tropicalis* and *C. glabrata*) [97,98]. Additionally, changes in other taxa have been identified, including an increase in the *Cystofilobasidiaceae* family and a decrease in *Leptosphaeria* and *Trichosporon* [95,99].

In IBD, fungal dysbiosis is globally characterized by a reduction in mycobiome diversity and an increase in the *Basidiomycota/Ascomycota* ratio [100,101]. Specifically, a decrease in *Saccharomyces cerevisiae* and an increase in *Candida albicans* have been observed [100,101]. In contrast, in pediatric IBD, *S. cerevisiae* has shown increased abundance alongside other yeasts, such as *Clavispora lusitaniae*, *Candida utilis*, *C. albicans*, and *Kluyveromyces marxianus*, which have been correlated with greater disease severity [100].

## 3. The Gut Microbiome in Colorectal Cancer

**Figure 2:**
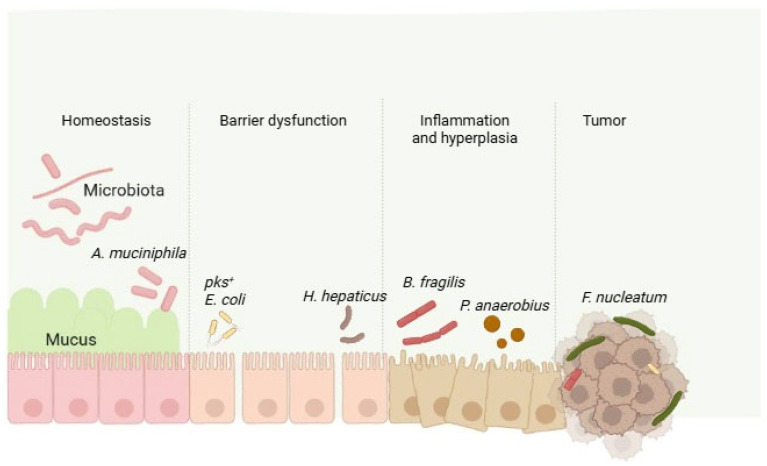
Alterations in the intestinal microbiota of patients with CRC are characterized by an imbalance in microbial composition, with an increase in pathogenic bacteria and a decrease in beneficial microbes, which may contribute to tumorigenesis (modified figure derived from [107] and created in https://BioRender.com).

Colorectal cancer (CRC) is one of the most common cancers in the world, with high rates of incidence and mortality, and it results from a combination of host genetic and environmental factors. Although some sporadic mutations may contribute to the development of CRC, environmental factors such as lifestyle, dietary habits, or age constitute the main triggers of the disease. Epidemiological studies show that IBD status represents a risk factor for the development of CRC. As has been reported in IBD, alterations in intestinal microbiota have been reported in CRC patients and, indeed, this may contribute to CRC development in IBD patients [102]. Animal research studies have demonstrated that the specific microbes associated with CRC may interact with epithelial or immune cells in the gut and promote tumorigenesis, including *Fusobacterium nucleatum* [103], enterotoxigenic *Bacteroides fragilis* [104], or *Peptostreptococcus anaerobius* [105] (Figure 2). Molecular mechanisms have also been described for those fungal species contributing to CRC pathogenesis [106].

### 3.1. Bacterial Markers in CRC

Earlier microbiome research studies using both fecal and mucosal samples have identified alterations in the bacterial microbiota associated with CRC patients [108,109,110]. Recent meta-analysis studies involving larger and multiple cohorts of patients have contributed to identifying bacterial signatures strongly associated with the CRC microbiome. These studies have served to validate previously described bacterial markers, such as *Fusobacterium nucleatum*, *Streptococcus bovis*, *Bacteroides fragilis*, *Porphyromonas asaccharolytica*, *Escherichia coli*, *Peptostreptococcus stomatis*, *Gemella morbillorum*, *Parvimonas micra*, *Solobacterium moorei*, and *Prevotella intermedia*, and establish them as potentially universal markers [9,111,112,113,114,115] (Table 2).

In addition, these studies have also discovered new bacterial markers. For example, a meta-analysis of fecal shotgun metagenomic data identified 29 species that were significantly enriched in CRC samples, including 11 newly described *Clostridiales* species belonging to the *Clostridiaceae/Lachnospiraceae* and *Ruminococcaceae* families [114]. A similar metagenomic study identified 62 depleted bacterial species in the CRC microbiome, including the novel beneficial *Clostridium butyricum*, *Streptococcus salivarius*, *Streptococcus thermophilus*, *Carnobacterium maltaromaticum*, and *Lactobacillus gallinarum* [113]. It is generally assumed that intestinal dysbiosis implies a decrease in bacterial diversity. However, Thomas AM et al. [115] found greater bacterial richness and abundance in the CRC microbiome compared to control samples, probably due to the presence of possibly oral bacteria that are rarely found in the healthy gut. This meta-analysis also identified novel alterations in specific taxa in the CRC microbiome, including an increment in *Streptococcus tigurinus*, *Streptococcus dysgalactiae*, and three different *Campylobacter* species, or a decrease in the beneficial *Gordonibacter pamelae* and *Bifidobacterium catenulatum*.

Several microbiome studies have noted that some bacterial taxa linked to the CRC gut microbiome are associated with the oral cavity. In addition to the first described oral pathogen *F. nucleatum* [120], other CRC-enriched bacteria associated with the oral cavity include *P. micra*, *G. morbillorum*, *P. stomatis*, *P. asaccharolytica*, *S. moorei*, and other species belonging to other genera, such as *Anaerococcus*, *Streptococcus*, *Granulicatella*, *Prevotella*, *Faecalibacterium*, and *Rothia* [109,111,116,118]. These findings have led to the “oral microbiome” hypothesis, which proposes that aggressive bacteria from the upper respiratory tract colonize the gut and promote tumorigenesis, as supported by animal studies of disease. Indeed, oral bacteria seem to be preferentially located in the mucosa of the CRC gut [118], where they may impact pathogenesis and may serve as potential biomarkers [121,122].

Further supporting this connection, recent research has investigated the oral microbiome’s potential for CRC screening by profiling microbiota from oral swabs, colonic mucosa, and the stools of individuals with CRC or polyps and controls [123]. Oral taxa like *Streptococcus* and *Prevotella* spp. were differentially abundant in CRC samples. An oral swab microbiota model distinguished CRC/polyp individuals from controls, with 96% specificity. Combining oral and fecal microbiota data increased analysis sensitivity, suggesting the oral microbiome’s value in non-invasive CRC detection. Shared bacterial networks between the oral and colonic microbiota were identified, and specific Lachnospiraceae species showed a potential protective role against CRC.

In further support of the oral microbiome’s role in colorectal carcinogenesis, another study investigated the association between the oral microbiome and the development of CRC. Oral swab samples were collected from 161 patients with CRC, 34 patients with colorectal adenoma (CRA), and 58 healthy volunteers [124]. Oral microbiota composition and diversity, assessed using 16S rRNA sequencing, were significantly different among the three groups, with the CRA group exhibiting the highest diversity. Functional potential analysis revealed that the cell motility pathway was over-represented in the CRA and CRC cohort groups compared to healthy controls. Notably, a random forest model based on oral microbial markers effectively distinguished the colorectal tumor groups from healthy controls in both the discovery and validation cohorts. This research further suggests a potential association between oral microbiome dysbiosis and colorectal cancer, indicating that oral microbiota-based biomarkers may be helpful in predicting the risks of developing CRA and CRC.

Differences in microbiome structure have been observed across the different stages of CRC development, which may serve to differentiate disease status [109,117] (Table 2). For example, a dysbiotic microbiome has already been used to identify early-stage CRC, in addition to the strong enrichment of specific CRC biomarkers such as *Fusobacterium*, *P*. *stomatis*, and *P. asaccharolytica* [109]. Other bacterial markers, such as *C. symbiosum* [125], *Oscillospira*, and *Haemophilus* [126], may also identify earlier stages of CRC. The identification of microbial markers in stools that are discriminatory for adenoma may represent an important tool for earlier non-invasive diagnosis. Unfortunately, most of the microbiome studies have failed to discriminate with great accuracy adenoma patients from control patients [109,117] and may provide evidence that the adenoma-associated microbiome is not dysbiotic enough to represent an adequate method of discrimination. However, a metagenomic study has detected three bacterial species, *F. nucleatum*, *C. hathewayi*, and, markedly, *Lachnoclostridium*, which were significantly enriched in the fecal samples of patients with adenoma compared to controls [9].

Functional analyses derived from metagenomic studies have shown a microbial metabolic signature associated with CRC status [114,115], which is collectively characterized by an increase in the abundance of genes participating in protein degradation (mostly related to *Clostridiales*), fermentative metabolism, and gluconeogenesis, and a depletion in the abundance of genes from carbohydrate catabolism. Remarkably, enrichment in bai genes from bile acid metabolism [114] and trimethylamine biosynthesis genes from choline metabolism [115] was also identified in CRC patients.

Current studies highlight the importance of a liquid biopsy for analyzing the gut microbiome of CRC patients. Gut microbiome-associated metabolites in plasma and serum show significant potential for CRC detection, prognosis, and treatment monitoring. A recent study introduced an integrated approach using serum metabolomic analysis to differentiate CRC and adenoma patients from healthy controls. This method, which analyzes gut microbiome-associated serum metabolites, identified elevated levels of specific microbial metabolites, such as N,O-Bis-(trimethylsilyl)phenylalanine. These elevated levels were positively correlated with bacterial species like *Clostridiales bacterium* VE202-01 and *Erysipelatoclostridium ramosum*, suggesting tumorigenesis-associated microbiome reprogramming [127].

### 3.2. Non-Bacterial Markers in CRC

To date, very few studies have investigated the potential association between the gut virome and CRC pathogenesis [128,129,130]. A meta-analysis of shotgun metagenomic data showed alterations in the gut virome associated with CRC pathogenesis, characterized by an enrichment of bacteriophage richness, especially those invading CRC-associated bacteria, such as *F. nucleatum*, *B. fragilis*, or *E. coli* pks^+^, a strain that produces a genotoxin that potentially contributes to cancer development. The authors also identified specific viral markers that were highly enriched in the CRC virome, including *Orthobunyavirus*, a genus not previously related to human microbiota [130]. Another study concluded that the Torque teno virus species that were identified were determined to be members of the Anelloviridae family, and their corresponding functional genes contributed valuable new information regarding the process of CRC carcinogenesis [128].

With respect to the mycobiome, earlier studies found alterations in fungal communities in the gut mucosa of patients with colorectal adenoma and posited that bacterial–fungal interactions could contribute to CRC pathogenesis [106,131]. For example, a meta-analysis of fecal metagenomic data identified alterations in fungal composition in different cohorts of CRC patients. Specifically, the authors found a higher *Basidiomycota* to *Ascomycota* ratio and the depletion of *Sacharomyces cerevisiae*, a major component of human gut microbiota, in CRC patients compared to controls [132].

## 4. The Gut Microbiome in Celiac Disease

Celiac disease (CeD) is a global health disease that affects around 1–2% of the population in developed countries, which is caused by the intake of gluten in genetically predisposed individuals, based on the expression of human leukocyte antigen (HLA)-DQ2 or HLA-DQ8 molecules. In humans, dietary gluten is partially metabolized by digestive enzymes, inhibiting the generation of the large peptides that are able to trigger adaptive immune responses in susceptible people who have lost their oral tolerance to gluten [133]. It is considered a systemic disorder that is characterized by a plethora of possible gluten-derived signs and symptoms, as well as disease-specific enteropathy. Genetic susceptibility is necessary but is not sufficient to trigger CeD, evidencing the contribution of not only other genetic but also non-genetic factors [134]. Most infectious agents, such as viruses, bacteria, fungi, and parasites, can induce autoimmunity via different mechanisms [135].

In contrast to IBD or CRC, our knowledge of the microbiome associated with CeD is still scarce, and more studies involving larger and multi-cohort individuals are required. Two main aspects are particularly challenging when characterizing the microbiomes associated with small-bowel diseases, which are basically related to sample obtention. In microbiome studies, the use of stools facilitates the process, but the extremely high microbial abundance in more distal parts of the gastrointestinal tract masks the resident microbiota in the small bowel [136]. In consequence, characterizing the microbiome associated with small bowel diseases relies on biopsy samples. However, biopsies contain larger amounts of human DNA and impede successful metagenomic sequencing; therefore, the identification of microbial taxa must be made at a higher resolution level, in addition to recording functional disruption.

### 4.1. Bacterial Markers in CeD

**Figure 3:**
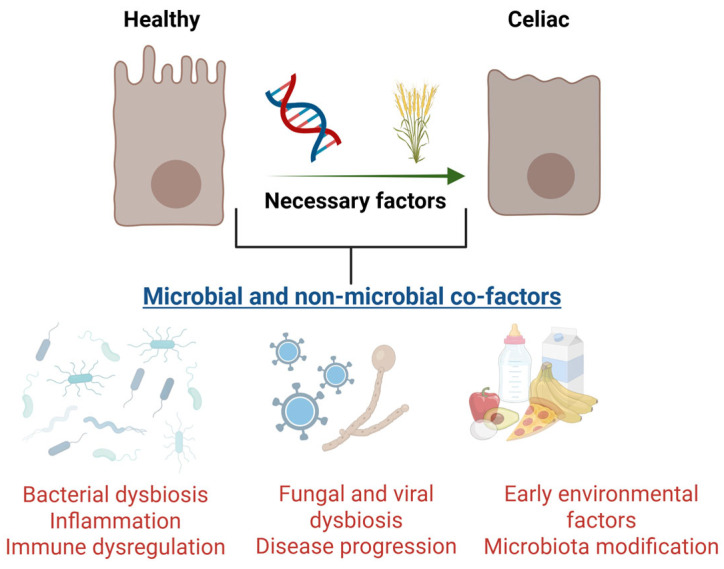
Genetic predisposition (HLA-DQ2/DQ8) and the presence of gluten are necessary factors for developing celiac disease (CeD). However, environmental factors, including microorganisms, play a significant role in the pathogenesis of celiac disease. Changes in the microbiome, such as a reduction in beneficial bacteria (such as *Lactobacillus and Bifidubacterium)* and an increase in pathogenic microorganisms (either bacterial, such as *Proteobacteria*, viral, such as Enterovirus and Reovirus, or fungal, such as *Candida* or *Saccharomycetes)*, are linked to disease onset and persistence, even in patients following a gluten-free diet. Other environmental factors lead to microbiota modification, such as early-life feeding patterns, including breastfeeding or the method of birth (cesarean section vs. vaginal delivery). Created in https://BioRender.com.

Microbial composition, both fecal and duodenal, is highly affected by CeD, both at the onset of the disease and when symptoms persist despite a gluten-free diet (GFD) [137]. It has been evidenced as a potential contribution of CeD-associated microorganisms to pathogenesis in humans. To date, it is known that the CeD-associated microbiome is generally characterized by a reduction in beneficial bacteria or probiotics, such as *Lactobacillus* or *Bifidobacterium*; other pathogenic bacteria that may be contributing to inflammation appear increased in number, such as some *Bacteroides* [138]. In many cases, dysbiosis persists, even on a GFD [139]. Moreover, duodenal bacteria such as *Pseudomonas aeruginosa* and *Lactobacillus* spp. participate in gluten catabolism and may define the magnitude of gluten-induced adaptive immune responses in CeD patients [133]. Duodenal *Lactobacillus* spp. are also able to metabolize and attenuate the inflammatory effect of the amylase-trypsin inhibitors (ATIs) [140] that are part of the non-gluten fraction of wheat grain; these activate innate immune responses and contribute to gluten-induced enteropathy [141] (Figure 3).

Several studies have been aimed at identifying the bacterial markers of CeD by analyzing duodenal biopsies or fecal samples. One recent study collecting fecal samples from children (ages 1, 2.5, and 5 years old) concluded that some metabolic pathways among the gut microbiota are identifiable years before a CeD diagnosis [142]. Other authors observed that *Firmicutes* were elevated in CeD progressors at the age of 1 year old, suggesting early microbial imbalance, whereas other taxa were significantly different compared to control samples (with no CeD progressors), with these possibly being involved in promoting intestinal permeability or an increased immune response to specific bacteria [143]. Consequently, some inflammation-associated metabolites were found in CeD progressors that showed correlations with some bacteria, such as a decrease in short-chain fatty acids or the production of certain bacterial compounds that might contribute to pathogenesis [144]. In contrast, a previous study showed no significant differences in the fecal microbiota composition between children who later developed CeD and those control children who did not [145]. Another article proposes a link between CeD genetic risk and the gut microbiota, which has been proposed to influence CeD development. The authors characterized the microbiomes of the first-degree relatives (FDRs) of CeD patients, which may provide an opportunity to predict disease risk since FDRs are susceptible to developing CeD [146]. Other authors analyzed the fecal microbiota from newborns with at least one FDR and found that those who later developed CeD showed decreased bacterial diversity and an increase in *Firmicutes* families, compared to those who did not develop the disease [147]. Furthermore, *Bifidobacterium longum* was associated with healthy children, whereas *Bifidobacterium breve* and *Enterococcus* species were associated with CeD [148]. At the level of the amplicon sequence variant (ASV), the mucosal microbiota of FDRs was characterized according to increment in the *Parvimonas*, *Granulicatella*, *Gemella*, *Bifidobacterium*, *Anaerostipes*, and *Actinomyces* genera [146] (Table 3).

There are several mechanisms linking microbiota to gluten-related disorders and, more specifically, to CeD [138]. Microbiota play a role in the regulation of gut barrier function, including gut permeability, immunity, and antigen-specific immune responses. Moreover, gluten metabolism and the immune response are highly affected by microbiota. As mentioned before, the abundance of some groups of beneficial bacteria, such as the probiotics *Lactobacillus* and *Bifidobacterium* spp., is diminished in the mucosa of celiac patients compared to controls [149,150]. In addition, studies have frequently observed an increase in aggressive taxa belonging to the Proteobacteria in both children and adults [151,152], a pathogenic phylum that may have a key role in CeD pathogenesis. Proteobacteria represent the most abundant phylum in duodenal biopsies from active CeD patients [153], which correlates with increased proteolytic activity against gluten substrates [154]. Proteobacteria have also been associated with clinical manifestations in active CeD patients [155], and an increased abundance of this phylum was also detected in the mucosa of CeD patients under a GFD and normal histology with persistent symptoms [156]. Considering that *Proteobacteria* and *Lactobacillaceae* (*Firmicutes* phylum) are core bacterial taxa in the small bowel [157], these findings suggest that the taxonomic and functional structure of CeD microbiota in the small bowel is profoundly altered.

**Table 3:** Main studies performed for the genomic determination of the microbial markers of celiac disease.

Study	Complexity of Study	Sequencing Method	Highlighted Bacterial Markers	
Costigan et al., 2024 [139]	One cohort with CeD before and after a GFD N = 36 fecal samples. One cohort HVs * N = 36 fecal samples.	Shotgun	CeD microbiota	
			Before GFD	After GFD (12 months)
			Increased vs. control *Escherichia coli*, *Enterobacter*, and *Peptostreptococcus*	Increased *Blautia wexlerae* Reduced Bifidobacteria
Kelley et al. (2025) [142]	One cohort of CeD progressors. Children and one cohort of age-matched healthy children (1–5 years old). N = 5–16 fecal samples.	16S	Microbial markers in CeD progressor at 1 year old	
			Increased: *Firmicutes*, *Ruminococcus bromii*, *Dialister invisus*, *Bifidobacterium dentium*, *Clostridium*, *Lachnospiraceae*, *Alistipes*, and *Faecalibacterium prausnitzii.*	Under-representation of *Lactobacillus* or *Eubacterium.*
			Altered microbiota years before the onset of CeD.	
Rentala et al. (2017) [145]	One cohort of non-CeD children with a high genetic risk of CeD. N = 27 fecal samples.	16S	Fecal microbiota composition differences between	
			Children who later developed CD	Children without disease or associated autoantibodies
			No statistically significant	
Bodkhe et al. (2019) [146]	One cohort of non-GFD CeD patients (Marsh > 2) (N = 23); one cohort of FDRs of CeD patients (N = 15), and one no-CeD control cohort (N = 24). Duodenal biopsy and fecal samples.	16S	FDR patients showed	
			Over-representation: *Parvimonas*, *Granulicatella*, *Gemella*, *Bifidobacterium*, *Anaerostipes*, and *Actinomyces.*	Under-representation: *Akkermansia*, *Dorea*, *Lactobacillus*, and *Haemophilus.*
El Mouzan et al. (2022) [147]	One cohort of children with CeD (N = 40), one cohort with healthy (N = 20), and non-CeD children (N = 19). Mucosal and fecal samples.	Shotgun	CeD children’s mucosa	CeD/non-CeD stools
			**Over-representation**: *Bifidobacterium angulatum* and *Roseburia intestinalis.*	169 bacterial species with significantly different abundances between individual types.
Olivares et al. (2018) [148]	One cohort of full-term newborns with at least one first-degree relative with CeD (N = 127). Fecal samples.	16S	Pathogenic bacteria in newborns with a high risk of CeD.	
			Increased: *Clostridium perfringens*, *Clostridium difficile*, and *Escherichia coli.*	
Sanchez et al. (2013) [152]	One cohort of active CeD patients (N = 32), non-active CeD patients (N = 17), and controls (N = 8). Duodenal biopsy.	16S	Active CeD children	
			Increased: Proteobacteria *Enterobacteriaceae*, *Staphylococcaceae (Klebsiella oxytoca*, *Staphylococcus epidermidis*, and *Staphylococcus pasteuri)*	Decreased: Firmicutes and *Streptococcaceae.*
D’Argenio et al. (2016) [153]	Cohorts of active CeD adults (N = 20), CeD adults in GFD (N = 6), and controls (N = 16). Duodenal biopsy.	16S	Active CeD patients	
			Increased: *Proteobacteria*, Neisseria genus (*Neisseria flavescens* was the most abundant *Neisseria* species in the duodenum).	Decreased: *Firmicutes* and *Actinobacteria.*
Wacklin et al., 2014 [156]	CeD patients in GFD with (N = 18) or without persistent symptoms (N = 18).	16S	GFD-treated patients with persistent symptoms	
			Increased: Proteobacteria (*p* = 0.04)	Decreased: Bacteroidetes and Firmicutes (*p* = 0.05). Microbial richness.

* HVs—healthy volunteers. GFD—gluten-free diet. FDR—first-degree relative.

Environmental factors also impact microbial populations and CeD pathogenesis. Breastfeeding may have a protective role in CeD development, due to its immunomodulatory properties. According to an interesting study, formula-fed newborns showed a higher diversity of Bacteroides species compared to breast-fed infants [158]. A similar study revealed that breastfeeding promoted the growth of beneficial *Clostridium leptum*, *Bifidobacterium longum*, and *B. breve*, and the depletion of some aggressive (*B. fragilis* and *E. coli*) and beneficial (the *Clostridium coccoides*-*Eubacterium rectale* group and *B. lactis*) taxa [159]. More recently, one study involving a larger cohort (n = 127) of newborns with at least one FDR showed that formula-milk-fed infants presented a higher prevalence of the aggressive pathogens *Clostridium perfringens* and *C. difficile* [148]. Despite the potential influence of milk feeding practices on the microbiota composition of high-risk infants, there are already some studies, including meta-analyses, demonstrating that although breastfeeding affects the gut microbiota and may even delay CeD onset, there is no conclusive evidence about a potential reduction in CeD risk [160,161]. A recent study investigated the composition of breast-milk microbiota and its potential influence on CeD development and indicated that certain bacterial strains in breast milk could shape the gut microbiota in early life, potentially affecting CeD risk [162].

Another environmental factor that plays a role in shaping microbial composition is diet, especially early-life feeding patterns, which can have long-term implications. Thus, Leonard and collaborators [163] analyzed the influence of genetic and environmental risk factors on the composition of the intestinal microbiota before solid food and gluten introduction in infants known to be at risk of CeD. Infants who were genetically predisposed to CeD displayed decreased numbers of several *Coprococcus*, *Streptococcus*, *Parabacteroides*, and *Veillonella* species and *Clostridium perfringens* at 4 and 6 months of age. This result was similar to that reported by Hov and colleagues, who also identified a lower abundance of *Coprococcus* and *Enterorhabdus* in individuals who were genetically at risk of developing different autoimmune disorders [164]. Regarding the type of delivery, the association of cesarean sections with decreased levels of *Bacteroides vulgatus* and *Bacteroides dorei* compared to vaginal delivery strengthens the possibility of the birth method influencing CeD risk.

### 4.2. Non-Bacterial Markers in CeD

Viral and fungal dysbiosis have also been described in CeD, evidencing specific taxa alteration. Regarding the role of viruses in the increase of CeD risk, a study investigated the virome in infant fecal samples and found a synergy between frequent exposure to enterovirus in earlier stages of life and a higher gluten intake [165]. Thus, children at the age of 1 to 2 years who were frequently exposed to enterovirus had a higher risk of developing CeD, suggesting that cumulative infections had a role in triggering the disease. This risk increased up to eightfold in children who consumed more gluten compared to the controls. The authors confirmed that enteroviruses, reoviruses, and rotaviruses may play a role in triggering celiac disease, while adenoviruses may have a protective effect. This finding supported the results from previous studies reporting the putative relationship between CeD and viral infections [166,167]. Other studies have highlighted the importance of *Reovirus* infection in gluten sensitivity and the breakdown of oral tolerance in susceptible individuals [168,169].

As yet, there have been no studies focused on the characterization of the specific virome of the small intestine (biopsies) in CeD patients, probably due to the methodological difficulties inherent to sample collection and, specifically, to the low input of the virus. However, evidence demonstrates that the potential link between viruses and autoimmune diseases such as CeD is, most importantly, supported by the scientific community [135].

Fungal microbiota communities have rarely been characterized in patients with CeD, although some researchers have reported that there is fungal dysbiosis in children with new-onset CeD [170]. Other authors described gut fungal profiles that suggested a role in the disease’s pathogenesis, showing more abundant taxa in samples from children with CeD, including *Tricholomataceae*, *Saccharomycetaceae*, *Saccharomycetes*, *Saccharomyces cerevisiae*, and *Candida*, whereas less abundant taxa included *Pichiaceae*, *Pichia kudriavzevii*, *Pneumocystis*, and *Pneumocystis jirovecii* [170]. Previously, other authors aimed to characterize the mycobiome associated with CeD, with the opposite result. One study revealed no differences in fungal richness or composition between duodenal biopsies from CeD patients and controls, and *Candida* species were barely present in both controls and CeD patients [171]. In contrast, another study found a higher abundance of *Candida* and *Saccharomyces* species in the stools of CeD patients compared to those from the controls [172]. Aerobic flora might contribute to a reduction in oxidative stress [173]. This finding indicates that alterations in the intestinal flora may induce pathology by modifying the redox state in the small intestine, potentially activating the immune system through oxidative agents [174]. Nevertheless, it cannot be concluded that this effect is solely attributable to changes in the redox state, as microRNA regulation also plays a role in intestinal signaling and the development of CeD [173]. Therefore, these disorders are associated with both the redox state and its signaling, mediated by microRNA, with alterations in the redox state potentially being induced by intestinal flora or other regulatory factors.

## 5. Advantages and Limitations of Sequencing Methodologies in Microbiome Studies

Next-generation sequencing (NGS) has become the predominant methodology in microbiome studies in recent years. Taxonomic profiling of the gut microbiota has been primarily conducted using either shotgun metagenomic sequencing or 16S rRNA amplicon sequencing, as detailed in Table 1, Table 2 and Table 3 of this review [175]. 16S rRNA sequencing targets the 16S ribosomal RNA subunit gene (16S rDNA), which contains conserved regions across bacterial species and hypervariable regions that are unique to specific genera. This approach has been extensively employed to characterize bacterial communities through amplification of the gene’s hypervariable portions. Consequently, numerous bioinformatic analysis pipelines have been developed over the past decade, encompassing steps for data preprocessing, quality control, taxonomic assignment, and community characterization [176]. In contrast, shotgun metagenomics involves the random sequencing of an entire sample’s metagenome, circumventing the need for specific primers and PCR amplicons, thereby reducing the biases associated with primer selection [177]. This sequencing strategy offers a more in-depth taxonomic characterization of microbial communities, enabling the detection of organisms from all domains. Furthermore, it yields complementary functional and structural genetic information. Metatranscriptomics, while less frequently used, has also been applied to investigate actively expressed genes within a microbial community, providing enhanced insights into target gene activity. It can serve as a valuable complement to shotgun metagenomics for elucidating the biological signatures expressed in various diseases [178].

The selection of an appropriate sequencing strategy is typically driven by the specific objectives of the researchers and clinicians [175,177]. Thus, 16S rRNA sequencing is often used for broad surveys of bacterial diversity, while shotgun metagenomics is preferred for functional studies or when strain-level resolution is required.

The main advantages and disadvantages of both strategies are summarized in Table 4.

## 6. Conclusions

The gut microbiome is recognized as a fundamental factor in the pathophysiology of various diseases, and specific alterations have a potential role as diagnostic tools or in therapeutic applications.

In IBD, microbiome analysis has facilitated the differentiation of disease subtypes, the assessment of treatment response, and the potential prediction of severe phenotypes. However, significant challenges persist regarding microbial classification and the absence of standardized protocols for sample processing.

In CRC, specific microbial markers, the associated metabolites, and non-bacterial elements demonstrate the potential for early detection, the investigation of carcinogenesis, and disease monitoring.

In CeD, bacterial, viral, and fungal dysbiosis is linked to immune disruption and inflammation, even with a gluten-free diet, although obtaining and analyzing microbiota samples from the small intestine present methodological hurdles [173,174].

Advances in sequencing and bioinformatics technologies have deepened the characterization of the microbiome, enabling the identification of global alterations and specific markers. Nevertheless, the characterization of viral and fungal genomes remains limited by the lack of comprehensive databases, potentially underestimating their prevalence. Furthermore, the high proportion of human DNA found in biopsies complicates metagenomic analysis. To address these limitations, future studies should integrate multi-omics approaches and employ and develop gnotobiotic animal models that mimic human pathogenesis to elucidate the specific contributions of microbial taxa.

Ultimately, while microbiome research has led to significant advances in understanding its role in various pathologies, multiple methodological and experimental challenges must still be overcome before these findings can be translated into effective clinical applications. Interdisciplinary collaboration and multicenter studies will be essential for standardizing protocols and harnessing the microbiome’s potential for disease prevention, diagnosis, and treatment.

## Figures and Tables

**Table 1:** The bacterial markers associated with IBD.

Study	Complexity of Study	Sequencing Method	Highlighted Bacterial Markers	
Gevers D et al., 2014 [41]	One cohort N = 668 biopsies and stools from pediatric CD (447) and controls (221).	Shotgun	Increased in CD vs. control	Decreased in CD vs. control
			*Enterobacteriaceae*, *Pasteurellaceae*, *Fusobacteriaceae*, *Neisseriaceae*, *Gemellaceae*, and *Veillonellaceae*	*Bifidobacteriaceae*, *Erysipelotrichaceae*, *Bacteroidales* (*Bacteroides*), *Clostridiales* (*Coprococcus*, *Ruminococcus*, *Roseburia*, *Blautia*, *Faecalibacterium*), including members from *Ruminococcaceae* and *Lachnospiraceae*.
Lewis JD et al., 2015 [42]	One cohort N = 112 stools from pediatrics CD (86) and controls (26).	Shotgun	Increased in CD vs. control	Decreased in CD vs. control
			*Escherichia*, *Klebsiella*, *Enterococcus*, and *Veillonella*	*Prevotella*, *Eubacterium*, *Odoribacter*, *Akkermansia*, *Roseburia*, *Parabacteroides*, *Alistipes*, *Coprococcus*, *Dorea*, and *Ruminococcus.*
Schirmer M et al., 2018 [33]	One cohort N = 405 biopsies and stools from new-onset pediatric UC patients.	16S	Increased in severe or moderate vs. mild CD	Decreased in severe or moderate vs. mild CD
			*Veillonela dispar*, *Aggregatibacter segnis*, *Campylobacter*, *Lachnospiraceae*, *Veillonela parvula*, *Haemophilus parainfluenzae*, and *Megasphaera* (all representing bacteria typical of the oral cavity).	*Ruminococcaceae* and *Lachnospiraceae* families.
Pascal V et al., 2017 [35]	Four discovery cohorts N = 178 stools from CD (34), UC (33), and controls (111).	16S	Increased in CD vs. control	Decreased in CD vs. control
			*Blautia*, *Escherichia*, *Fusobacterium*, *Dialister*, *Sutterella*, and *Collinsella.*	*Ruminococcus*, *Coprococcus*, *Roseburia*, *Ocillospira*, *Faecalibacterium*, *Lachnospira*, *Turicibacter*, *Clostridium*, *Parabacteroides*, *Anaerostipes*, and *Methanobrevibacter*
			Increased in CD vs. non-CD	Decreased in CD vs. non-CD
			*Fusobacterium* and *Escherichia*.	*Christensenellaceae*, *Peptostreptococcaceae*. *Faecalibacterium*, *Anaerostipes*, *Methanobrevibacter*, and *Collinsella*.
Vich Vila A, et al., 2018 [37]	Three discovery cohorts N = 1380 stools from CD (208), UC (126), IBD-unclassified (21), and controls (1025).	Shotgun	Increased in CD vs. control	Decreased in CD vs. control
			*Enterobacteriaceae*, *Lachnospiraceae*, *Bacteroidaceae*, *Streptococcaceae*, *Erysipelotrichaceae*, and *Eggerthellaceae*.	*Lachnospiraceae*, *Ruminococcaceae*, *Clostridiaceae*, *Eubacteriaceae*, *Oscillospiraceae*, *Erysipelotrichaceae*, and *Peptostreptococcaceae*.
			Increased in UC vs. controls	Decreased in UC vs. controls
			*Bacteroidaceae*.	*Lachnospiraceae*, *Ruminococcaceae*, *Clostridiaceae*, and *Peptostreptococcaceae*.
Franzosa EA et al., 2018 [36]	One discovery cohort N = 155 stools from CD (68), UC (53), and non-IBD control (34) patients. *Validation with one additional cohort N = 65 stools from CD (20)*, *UC (23)*, *and non-IBD control (22) patients.*	Shotgun	Increased in IBD vs. non-IBD	Decreased in IBD vs. non-IBD
			*Roseburia*	*Ruminococcus*, *Dorea*, *Eubacterium*, *Roseburia*, *Coprococcus*, *Faecalibacterium*, *Lachnospiraceae bacterium*, *Anaerostipes*, *Alistipes*, *Bacteroidales*, *Barnesiella*, *Oscillibacter*, *Holdemania*, *Subdoligranulum*, *Adlercreutzia*, *Gordonibacter*, and *Anaerotruncus*. The species *Roseburia hominis*, *Dorea formicigenerans*, and *Ruminococcus obeum* exhibited the strongest effects.
			Increased in UC vs. non-IBD:	Increased in CD vs. non-IBD:
			*Bifidobacterium breve* and *Clostridium symbiosum*	*Dorea*, *Lactobacillus*, *Pediococcus* (2), *Blautia*, *Clostridium*, *Ruminoccocus*, *Lachnospiraceae bacterium* (2), and *Escherichia* (2). The species *Ruminococcus gnavus*, *Escherichia coli*, and *Clostridium clostridioforme* exhibited the strongest effects.
Nishino K et al., 2018 [38]	One cohort N = 174 mucus samples from CD (26), UC (43), and non-IBD control (14) patients.	16S	Increased in CD vs. non-IBD	Decreased in CD vs. non-IBD
			Proteobacteria such as *Escherichia*, *Ruminococcus gnavus*, *Cetobacterium*, *Actinobacillus*, and *Enterococcus*.	*Faecalibacterium*, *Coprococcus*, *Prevotella*, and *Roseburia*
			Increased in CD vs. UC	Increased in UC vs. CD
			*Escherichia*, *Ruminococcus gnavus*, *Clostridium*, *Cetobacterium*, and *Peptostreptococcus*.	*Faecalibacterium*, *Blautia*, *Bifidobacterium*, *Roseburia*, and *Citrobacter*.
Yilmaz B et al., 2019 [39]	One discovery cohort N = 1255 biopsies from IBD and non-IBD control patients. *Validation with one additional cohort N = 1846 biopsies from IBD and non-IBD control patients*	16S	Increased in CD vs. non-IBD	Decreased in CD vs. non-IBD
			*Blautia*, *Ruminococcaceae*, and *Enterobacteriaceae*.	*Bifidobacterium*, *Collinsella*, Barnesiellaceae, *Butyricimonas*, *Rikenellaceae*, *Clostridiales*, *Lachnospiraceae*, *Coprococcus*, *Lachnospira*, *Roseburia*, *Ruminococcaceae*, *Faecalibacterium*, and *Erysipelotrichaceae*.
			Increased in UC vs. non-IBD	Decreased in UC vs. non-IBD
			*Bifidobacterium*, *Collinsela*, *Odoribacter*, *Blautia*, *Lachnospiraceae*, and *Ruminococcaceae*.	*Bacteroidetes*, *Parabacteroides*, *Clostridiaceae*, and *Tenericutes phylum*.
Lloyd-Price J et al., 2019 [8]	One cohort N = 1595 fecal samples from 130 CD, UC, and control patients.	Shotgun	Increased in CD vs. non-CD	Decreased in CD vs. non-CD
			*Escherichia coli*, *Ruminococcus torques*, and *Bacteroides fragilis.*	*Faecalibacterium prausnitzii*, *Roseburia hominis*, *Subdoligranulum*, *Bacteroides thetaiotaomicron*, and *Coprococcus comes*.
			Increased in UC vs. non-UC	
			*Ruminococcus gnavus* and *Veillonela*.	
Mevlut Ulas et al., 2024 [34]	One cohort N = 49 mucus samples from CD (13), UC (8), and non-IBD control (28) patients.	16S	Increased in CD and UC vs. non-IBD	
			*Faecalibacterium prausnitzii*, *Bacteroidales*/*Bacteroidota*, and *Gammaproteobacteria*	

Note: CD—Crohn’s disease; UC—ulcerative colitis.

**Table 2:** Bacterial markers associated with CRC.

Study	Complexity of Study	Sequencing Method	Highlighted Bacterial Markers	
Nakatsu G et al., 2015 [116]	One cohort N = 160 mucosal biopsies of adenoma and adenoma-adjacent mucosae (47), carcinoma and carcinoma-adjacent mucosae (52), and controls (61). *Validation with two additional previously published cohorts.*	16S	Increased in CRC vs. control	Decreased in CRC vs. control
			*Fusobacterium*, *Bacteroides fragilis*, *Parvimonas*, *Peptostreptococcus*, *Gemella*, and *Leptotrichia*	*Bacteroides*, *Blautia*, *Sutterella*, *Faecalibacterium prausnitzii*, *Collinsella aerofaciens*, and *Alistipes putredinis*
Feng Q et al., 2015 [117]	One cohort N = 156 fecal metagenomes from colorectal adenoma, carcinoma, and healthy controls.	Shotgun	Increased in CRC vs. advanced adenoma or control	Decreased in carcinoma or adenoma vs. control:
			*Bacteroides* and *Parabacteroides* spp., *Alistipes putredinis*, *Bilophila wadsworthia*, *Lachnospiraceae* sp., *Escherichia coli*, and oral anaerobes *Fusobacterium* sp., *Pavimonas micra*, *Gemella morbillorum*, and *Peptostreptococcus stomatis*.	*Bifidobactium animalis* and *Streptococcus thermophilus*.
Yu J et al., 2017 [111]	Two discovery cohorts N = 168 fecal metagenomes from CRC (90) and controls (78). *Validation with two additional previously published cohorts.*	Shotgun	Increased in CRC vs. control	Decreased in CRC vs. control
			*Parvimonas micra*, *Solobacterium moorei*, *Fusobacterium nucleatum*, and *Peptostreptococcus stomatis*.	*Eubacterium ventriosum*.
Flemer B et al., 2017 [118]	One cohort N = 179 fecal and/or mucosal metagenomes from patients with CRC (102), polyps (21), and healthy controls (56). *Validation with two additional previously published cohorts*	16S	Increased in CRC vs. control (mucosal)	
			*Bacteroides*, *Roseburia*, *Ruminococcus*, *Oscillibacter*, and the oral pathogens *Porphyromonas*, *Peptostreptococcus*, *Parvimonas*, and *Fusobacterium*	
Dai Z et al., 2018 [113]	Four cohorts N = 526 fecal metagenomes from CRC (255) and controls (271).	Shotgun	Seven CRC-enriched bacteria vs. control	Twenty CRC-depleted species with the largest fold change from a total of 69 CRC-depleted bacteria
			*Porphyromonas asaccharolytica*, *Fusobacterium nucleatum*, *Prevotella intermedia*, *Parvimonas micra*, *Bacteroides fragilis*, *Alistipes finegoldii*, and *Thermanaerovibrio acidaminovorans.*	*Ehrlichia ruminantium*, *Bartonella bacilliformis*, *Eubacterium eligens*, *Acinetobacter* sp. *ADP1*, *Mycoplasma canadense*, *Weissella cibaria*, *Dictyoglomus thermophilum*, *Thermosipho africanus*, *Thermodesulfobacterium geofontis*, *Campylobacter iguaniorum*, *Spiroplasma diminutum*, *Candidatus Phytoplasma australiense*, *Mycoplasma capricolum*, *Arcobacter* sp., *L Streptococcus* sp. *I−G2*, *Staphylococcus argenteus*, *Streptococcus thermophilus*, *SR1 bacterium RAAC1*, *Streptococcus salivarius*, and *Bifidobacterium catenulatum.*
Shah MS et al., 2018 [114,119]	Nine previously published cohorts N = 509 total fecal samples from CRC (195), colorectal adenoma (79), and controls (235)	16S	Increased in adenoma vs. control	Decreased in adenoma vs. control
			*Prevotella*, *Methanosphaera*, *Succinovibrio* species, *Haemophilus parainfluenzae*, and the strains of the *Synergistes* family DSM 25858 and *Methanosphaera stadtmanae* DSM 3091.	*Akkermansia muciniphila*.
			CRC increased bacteria vs. control	Increased in CRC and adenoma vs. control
			*Peptostreptococcus anerobius*, *Parvimonas*, *Porphyromonas*, *Akkermansia muciniphila*, *Fusobacterium* sp., *Parabacteroides distasonis*, *Streptococcus anginosus*, *Porphyromonas asaccharolytica* ATCC 25260, *Parvimonas micra* ATCC 33270, *Pantoea agglomerans*, and Proteobacteria.	*Ruminococcus*, *Lactobacillus*, and Enterobacteriaceae.
Wirbel J et al., 2019 [114]	Eight discovery cohorts N = 768 total fecal metagenomes from CRC (285) and control (290) from five cohorts. *Validation with three additional independent previously published cohorts comprising metagenomes from CRC (101) and controls (102*).	Shotgun	A core set of 29 species increased in CRC vs. control	
			*Parvimonas micra*, *Gemella morbillorum*, *Peptostreptococcus stomatis*, *F. nucleatum subspecies animalis*, *Dialister*, *Unknown Porphyromonas*, *Solobacterium moorei*, *Porphyromonas uenonis*, *Clostridium symbiosum*, *Clostridiales*, *Hungatella hathewayi*, *Prevotella intermedia*, *Porphyromonas somerae*, *Porphyromonas asaccharolytica*, *F. nucleatum subspecies nucleatum*, *Parvimonas species*, *Prevotella nigrescens*, *Porphyromonas*, *Ruminococcus torques*, *F. nucleatum subspecies vincentii*, *Fusobacterium species oral taxon 370*, *Peptostreptococcaceae*, *Anaerococcus obesiensis/vaginalis*, *Anaerotruncus*, *P. uenonis*, *Clostridiales*, *Porphyromonas*, *Clostridium bolteae/clostridioforme*, and *Subdoligranulum species*.	
Thomas AM et al., 2019 [115]	Nine discovery cohorts N = 969 total fecal metagenomes from CRC (313), adenoma (143), and control (308) from 7 cohorts. *Validation with two additional independent previously published cohorts comprising metagenomes from CRC (100) and controls (105).*	Shotgun	A core set of 26 species increased in CRC vs. control metagenomes	
			*Fusobacterium nucleatum*, *Parvimonas micra*, *Parvimonas* spp., *Gemella morbillorum*, *Peptostreptococcus stomatis*, *Solobacterium moorei***,** *Porphyromonas asaccharolytica*, *Clostridium symbiosum*, *Anaerococcus vaginalis*, *Prevotella intermedia*, *Bacteroides fragilis*, *Porphyromonas somerae*, *Anaerococcus obesiensis*, *Porphyromonas uenonis*, *Peptostreptococcus anaerobius*, *Streptococcus constellatus*, *Granulicatella adiacens*, *Methanobrevibacter smithii*, *Eikenella corrodens*, *Ruminococcus torques*, *Peptostreptococcus* spp., *Streptococcus gallolyticus*, *Methanobrevibacter* spp., *Actinomyces cardiffensis*, *Campylobacter ureolyticus*, and *Anaerotruncus* spp. Biomarkers enriched in the majority of the datasets are underlined.	
Liang JQ et al., 2019 [9]	Two cohorts N = 1012 fecal metagenomes from CRC (274), adenoma (353), and controls (385).	Shotgun	Increased in CRC or adenoma vs. control	
			*Fusobacterium nucleatum*, *Clostridium hathewayi*, and *Lachnoclostridium*.	

**Table 4:** Main advantages and disadvantages of shotgun sequencing and 16S rRNA sequencing as strategies for gut microbiome studies.

Sequencing Methodology	Advantages	Disadvantages
16S rRNA	- Efficient and cost-effective - Less host contamination - Lower risk of false positives - More user-friendly, with easier interpretation of the results	- It is subject to biases (hypervariable regions and primer-dependent PCR amplification) - Limited taxonomic resolution, often to the genus level or above - Limited predicted functional information
Shotgun	- Broader taxonomic coverage (species- and strain-level resolution) - More accurate functional profiling - Offers the possibility of detecting previously unknown species and strains of microbes	- More expensive - More complex bioinformatic analysis - More risk of false positives, due to host contamination
